# Supplementary material for: An Imidazo[1,5-a]pyridine Benzopyrylium-Based NIR Fluorescent Probe with Ultra-Large Stokes Shifts for Monitoring SO2
Source: Molecules. 2023 Jan 5;28(2):515. doi: 10.3390/molecules28020515 (PMC9863934; doi:10.3390/molecules28020515)
Supplement: Supplementary file 1 [file molecules-28-00515-s001.zip › molecules-2118318-supplementary_3rd proof.pdf]

Imidazo[1,5-a]pyridine-benzopyrylium based NIR fluorescent probe for monitoring SO_2 with ultralarge Stokes shifts

Renle Cui[1], Caihong Liu[1], Ping Zhang[1], Kun Qin[*], Yanqing Ge[*]

Department of Chemistry and Pharmaceutical Engineering, Shandong First Medical University & Shandong Academy of Medical Sciences, No. 619, Changcheng Road, Taian, Shandong 271016, China. E-mail: yqge@sdfmu.edu.cn

[1] equal contribution

Instruments

^{1}H NMR and ^{13}C NMR spectra were measured on a Bruker Avance 400 (400 MHz) spectrometer ($CDCl_3$ or DMSO-d_6 as solvent and tetramethylsilane (TMS) as an internal standard). High-resolution mass spectra (HRMS) were measured on an Agilent 6546 LC/Q-TOF spectrometer using electron spray ionization (ESI) technique. Chromatographic separations were done by column chromatography using 200-300 mesh silica gel. UV-vis spectra and fluorescence spectra were recorded on a UV-2600 spectrometer (Shimadzu) and FS-5 luminescence spectrophotometer (Edinburg) at room temperature and Fluorescence imaging was obtained by Olympus FV 3000.

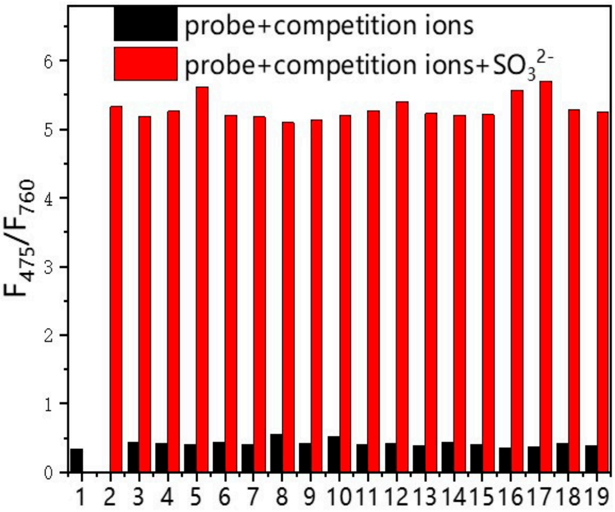

Figure S1. Ratiometric fluorescence responses F_{475}/F_{760} of **IPB-RL-1** upon the addition of 10 equiv. SO_3^{2-} in the presence of 100 eq. background ions (1, probe; 2, SO_4^{2-}; 3, Br^-; 4, ACO^-; 5, Cl^-; 6, ClO^-; 7, Cys; 8, ClO_4^-; 9, GSH; 10, F^-; 11, $H_2PO_4^-$; 12, HCO_3^-; 13, HPO_4^{2-}; 14, HS^- ; 15, I^- ; 16, NO_2^-; 17, NO_3^- ; 18, $S_2O_8^{2-}$; 19, SO_3^{2-}).

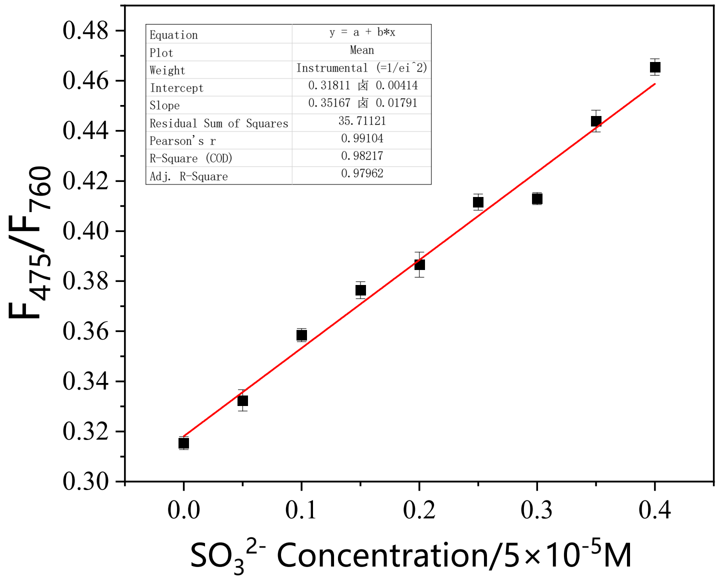

Figure S2. Relationship between fluorescence intensity ratio (F_{475}/F_{760}) and SO_3^{2-} concentration.

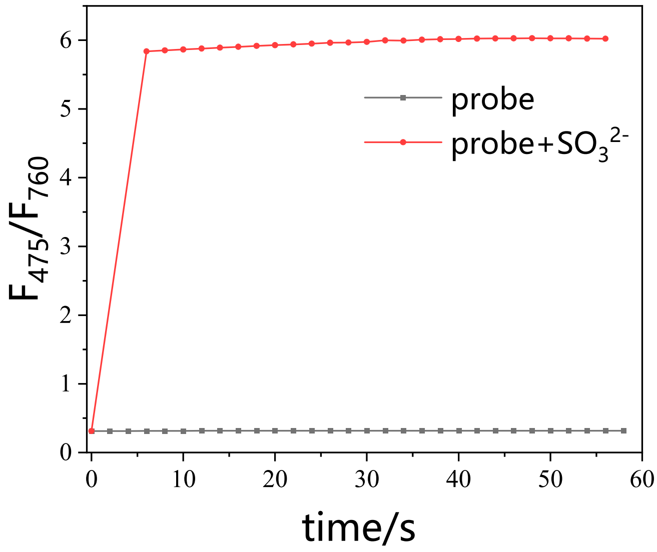

Figure S3. Time dependent increase of **IPB-RL-1** fluorescence intensities after addition of SO_3^{2-}.

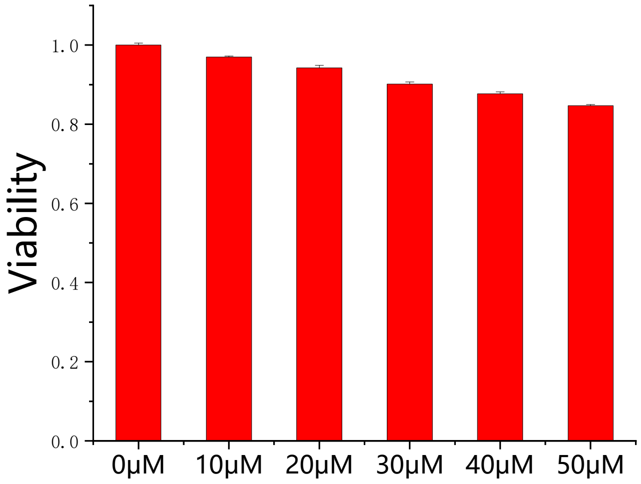

Figure S4. Cytotoxicity of **IPB-RL-1**.

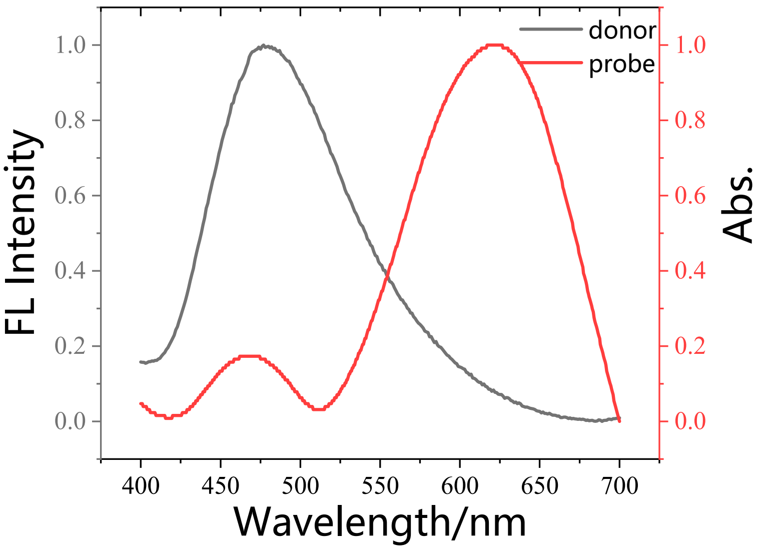

Figure S5. Normalized emission spectra of donor (compound **3**) and normalized absorption spectra of **IPB-RL-1**.

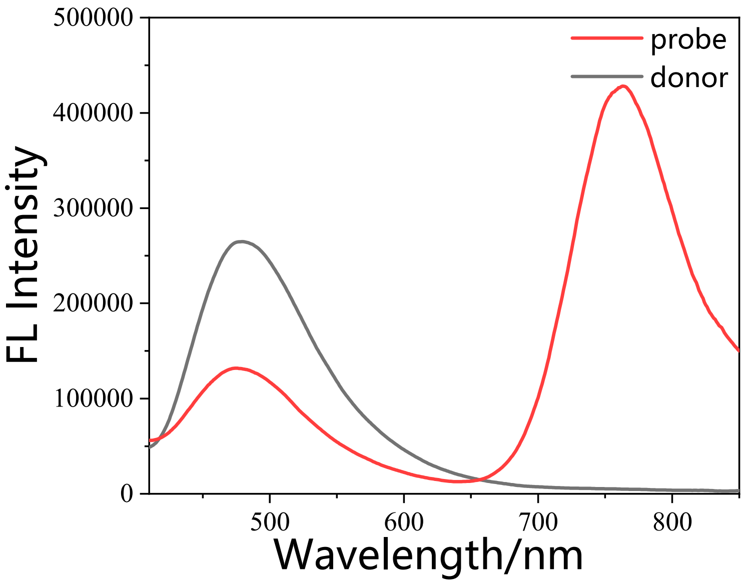

Figure S6. The emission spectrum of probe **IPB-RL-1** and donor.

Energy transfer efficiency = $1-F_{DA}/F_{D}$ = 51%

Where F_{DA} is the fluorescence intensity of the donor in the presence of the acceptor, F_{D} is fluorescence intensity of the donor in the absence of the acceptor.

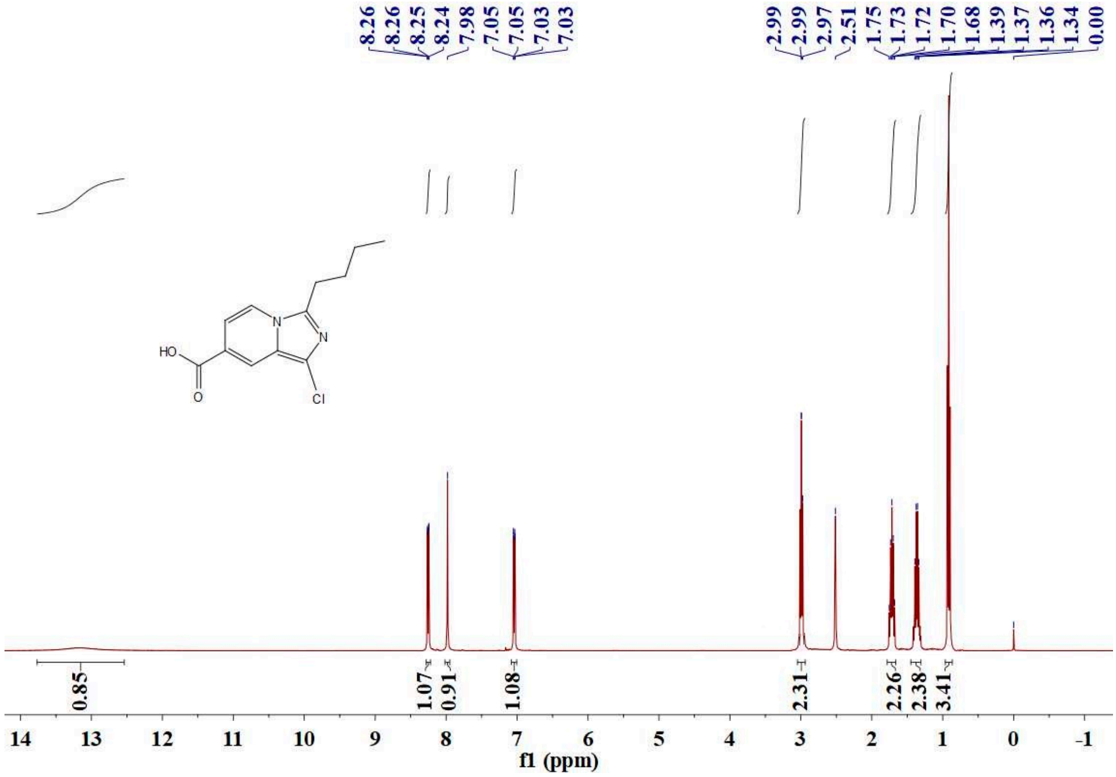

Figure S7. ^{1}H NMR of compound **1**.

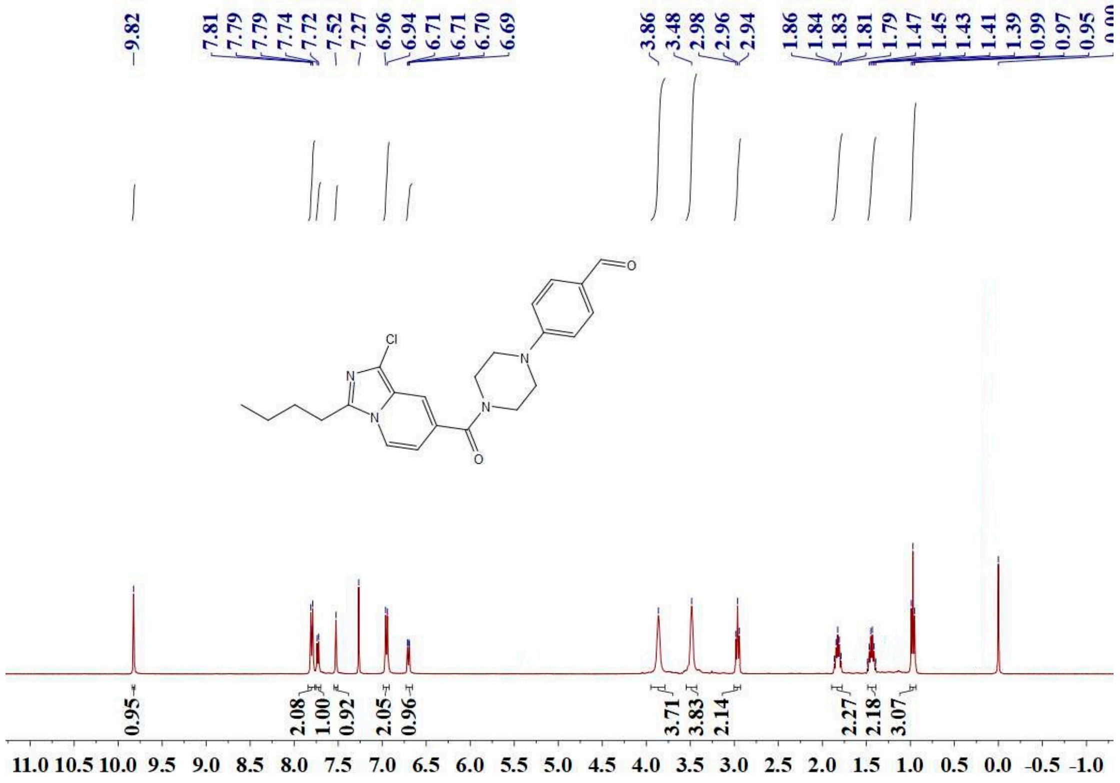

Figure S8. ^{1}H NMR of compound **3**.

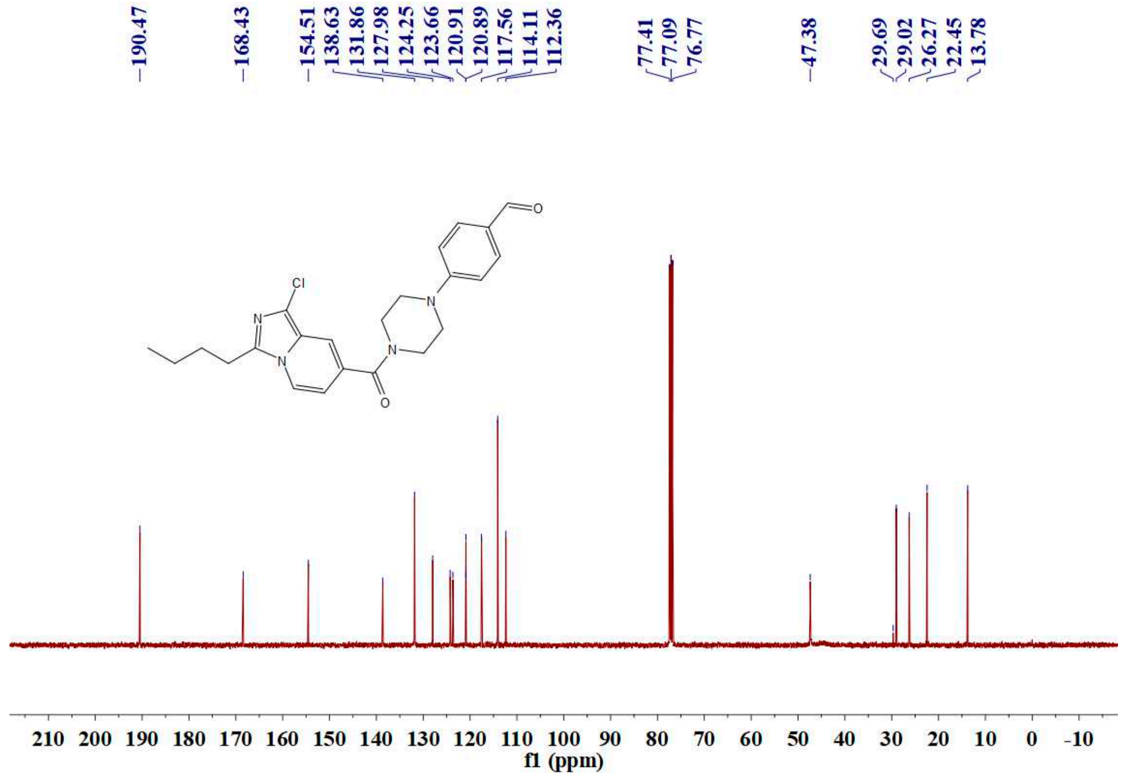

Figure S9. ^{13}C NMR of compound **3**.

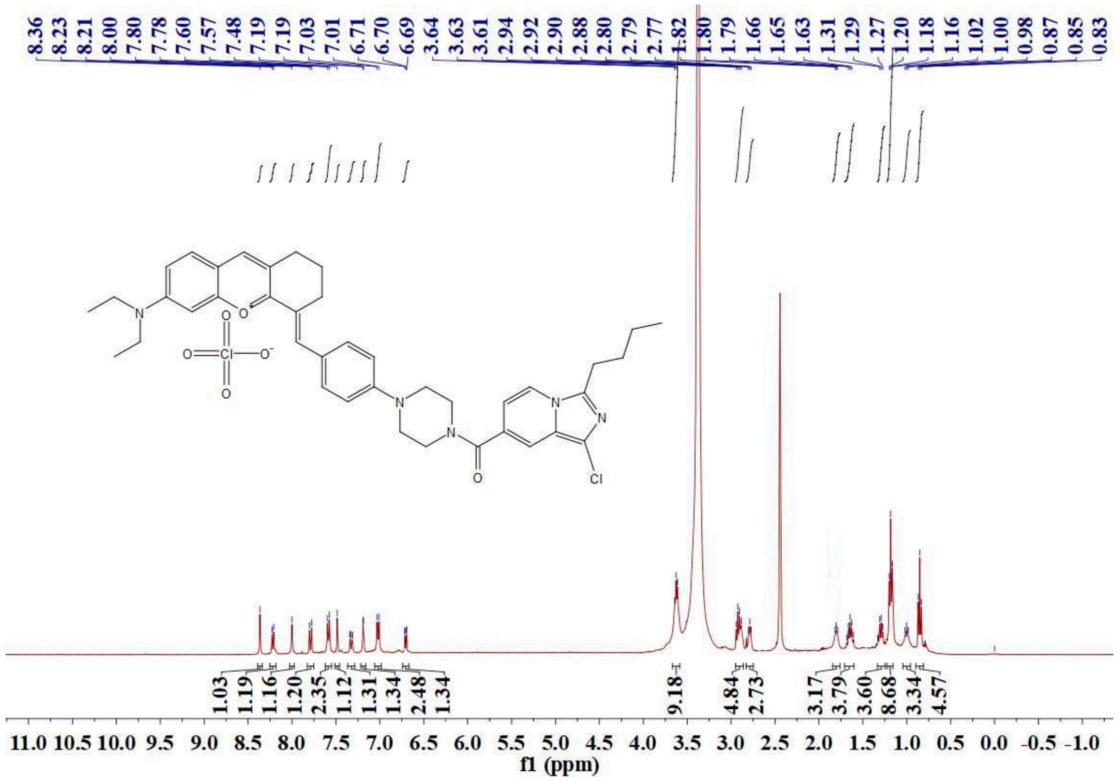

Figure S10. ^{1}H NMR of probe **IPB-RL-1**.

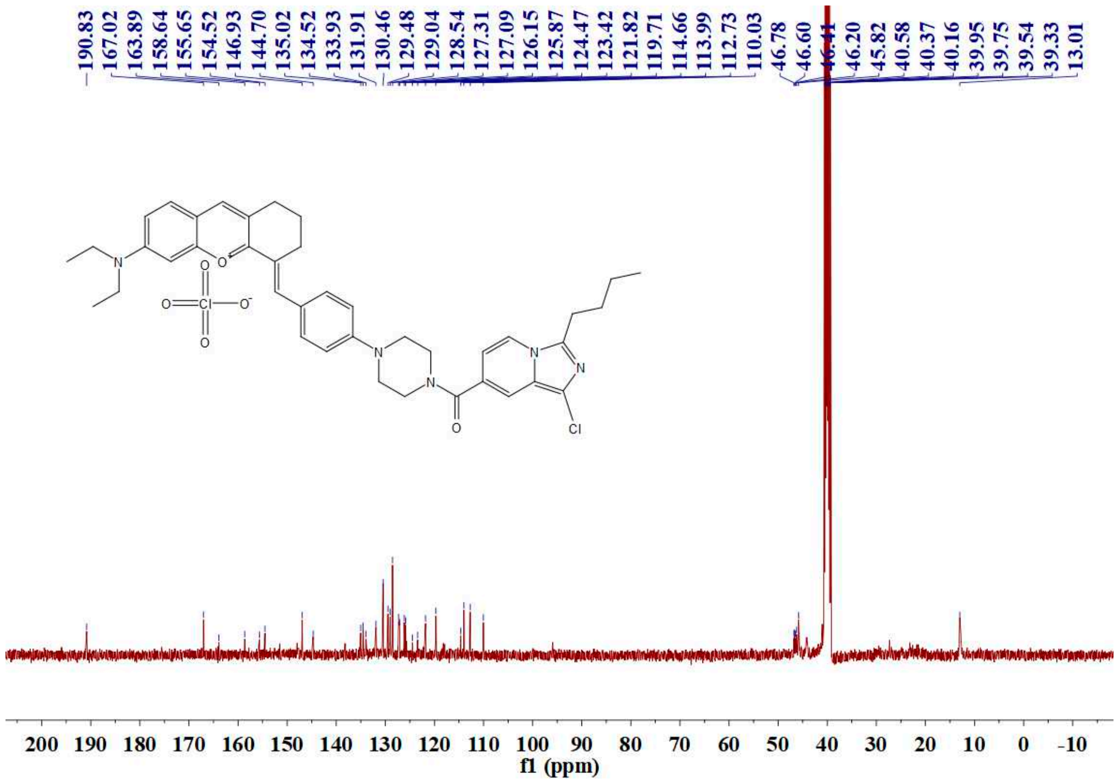

Figure S11. ^{13}C NMR of probe **IPB-RL-1**.

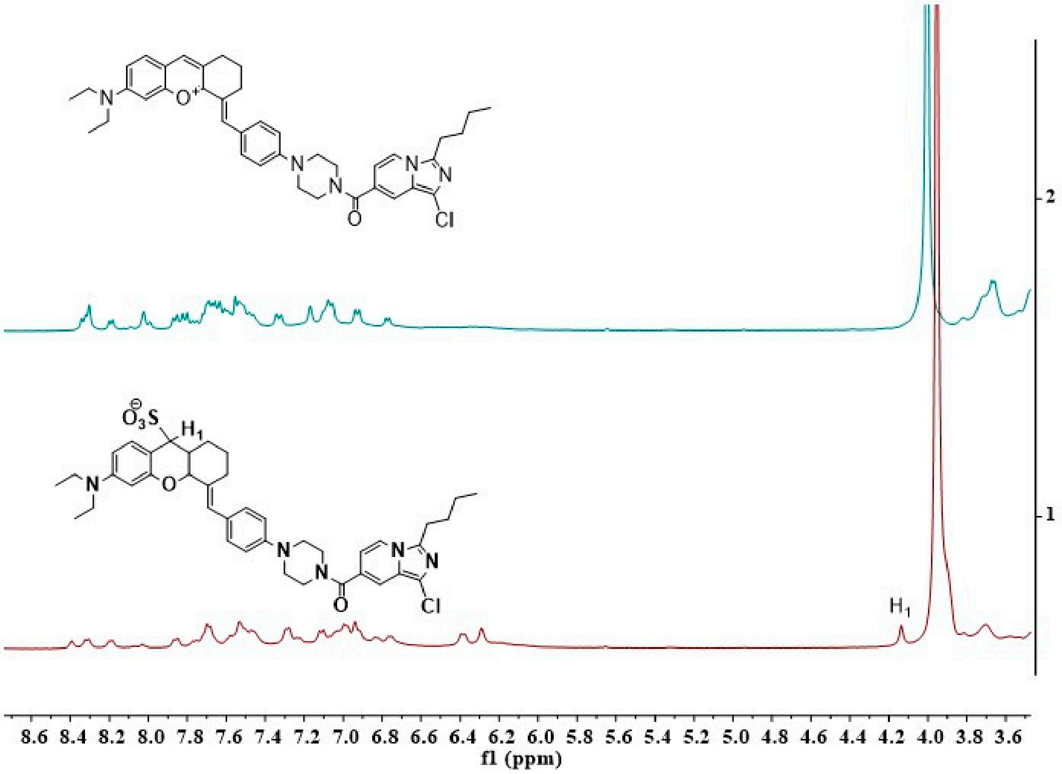

Figure S12. The ^{1}H NMR of **IPB-RL-1** in the presence of SO_3^{2-} in DMSO- d_6.

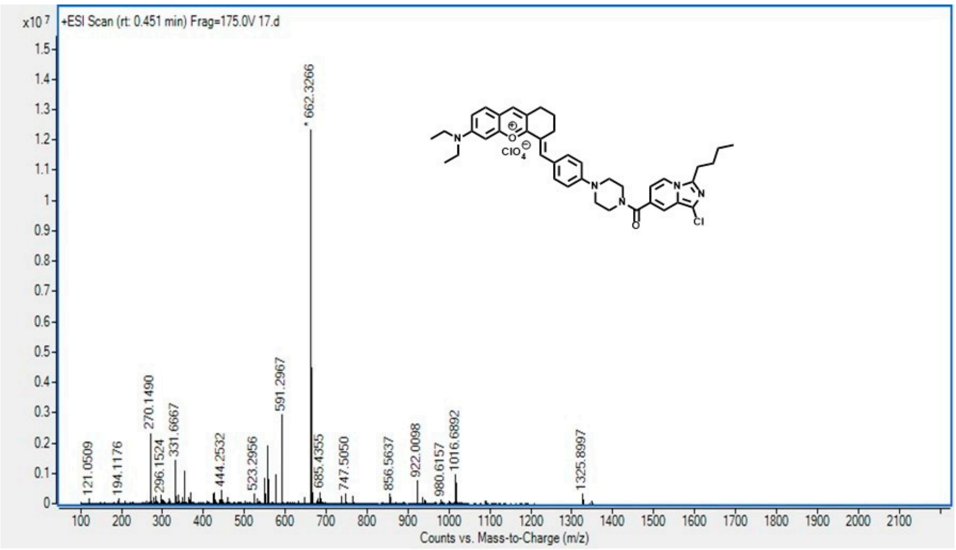

Figure S13. HRMS of probe **IPB-RL-1**.

Table S1. Comparison with other probes.

Probe	λ_{ex} (nm)	Stokes shift (nm)	LOD (nM)	response time	Ref.
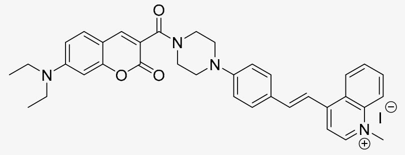	420	251	17	50min	31
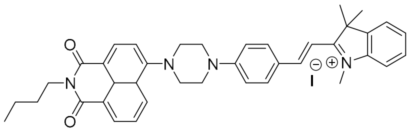	410	115	98.1	150s	32
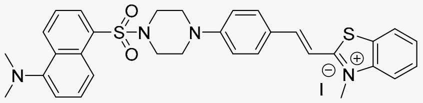	390	200	69		33

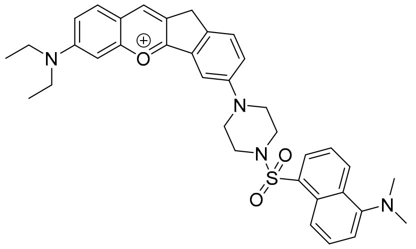	380	255	1.08	22s	34
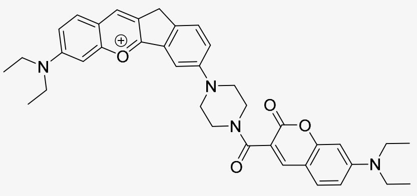	410	225	1.08	22s	34
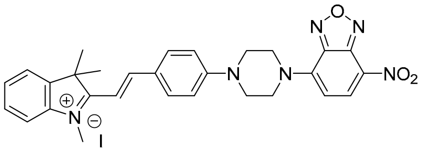	345	250	68	2min	35
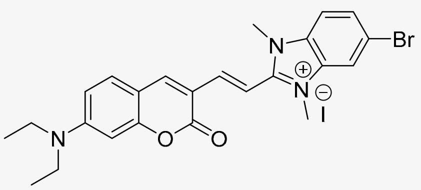	420	160	×	36min	36
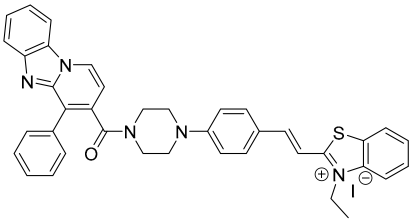	380	198	26.7	60min	28
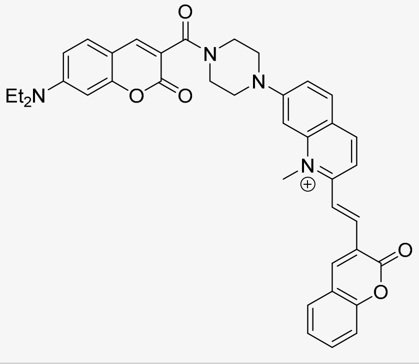	395	132	100	50min	25
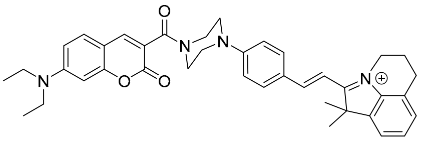	405	195	66000	5min	11

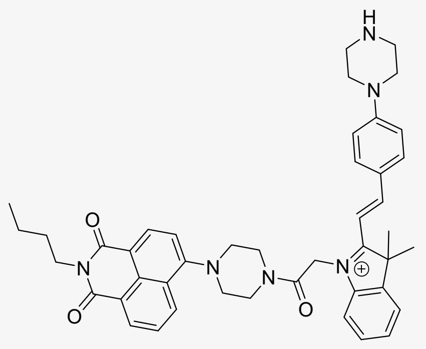	410	180	61.2	30s	37
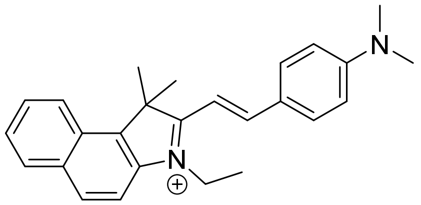	405	206	58.6	30s	38
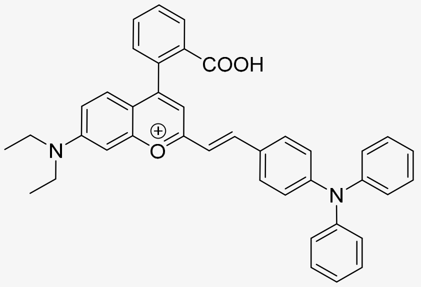	640	170	3200	80min	39
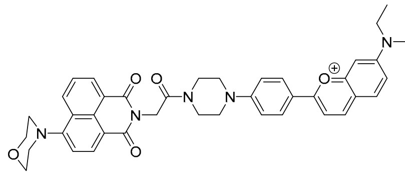	390	240	7.48	8s	16
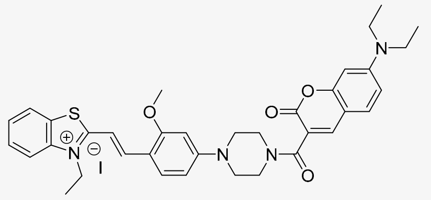	380	203	12.6	35s	40
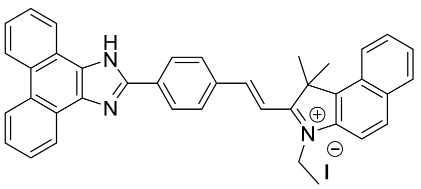	370	80	26	20s	41
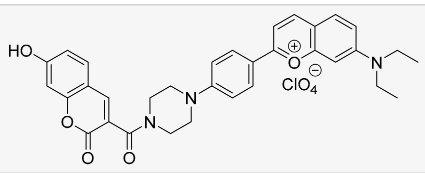	405	230	78	15s	42
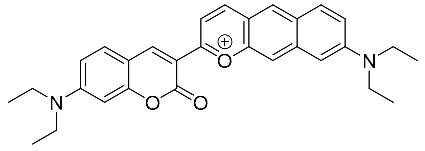	470	224	47	10s	43

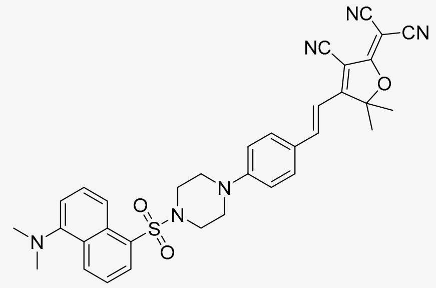	390	155	66	60min	22
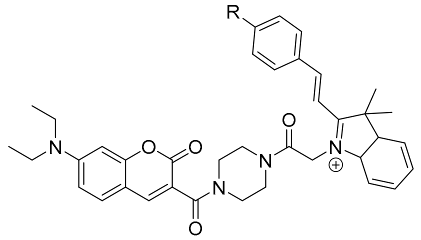	430	159	12.85	3min	44
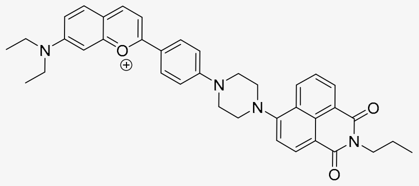	425	215	1210	5s	12
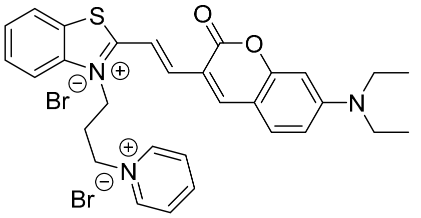	400	48	292	1min	45
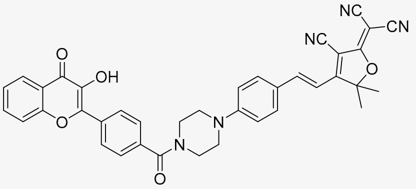	345	296	17	20min	46
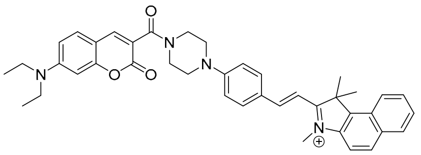	420	200	15.6	13min	47
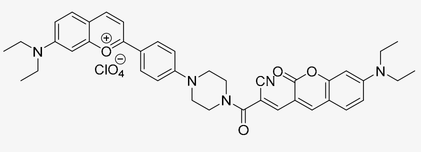	405	233	160	40s	20
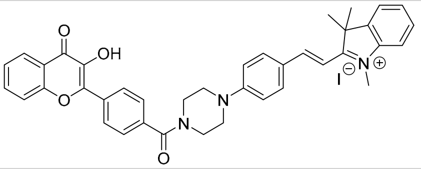	345	245	16	2min	48

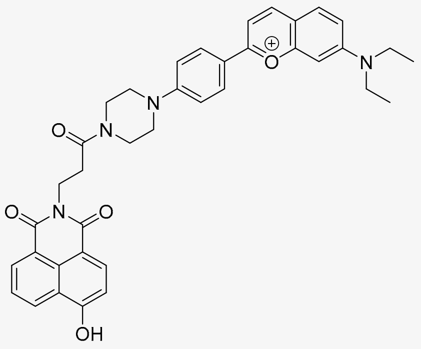	405	229	47	8min	49
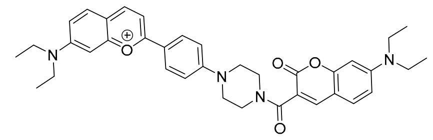	420	210	17.7	1min	19
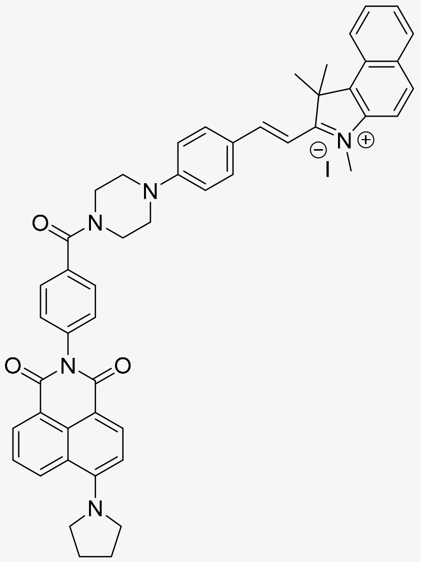	440	170	16.2	12min	50
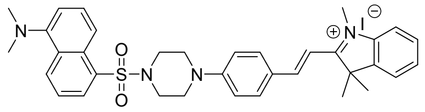	410	172	100	2min	51
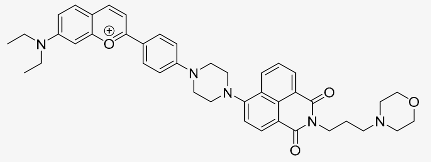	470	180	17	20s	21
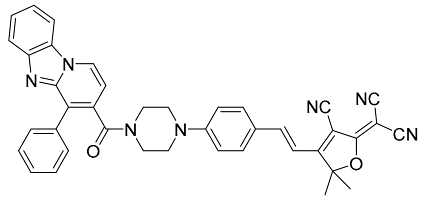	380	260	62	2min	52
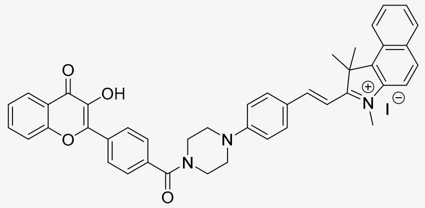	410	205	26000	10min	23

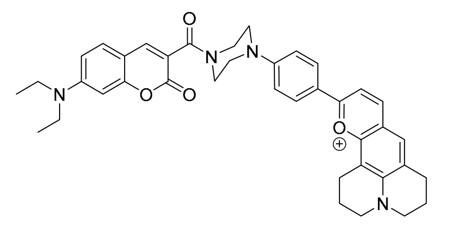	415	235	5910	1min	53
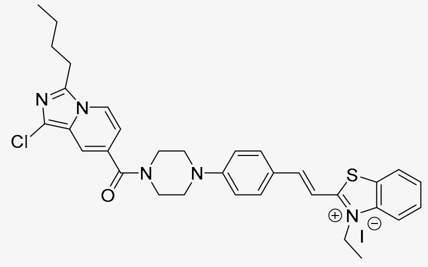	380	196	70	20min	54
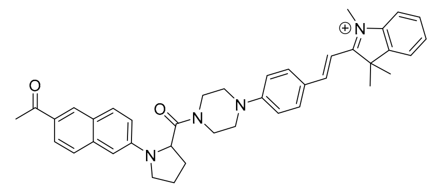	378	122	240	10min	17
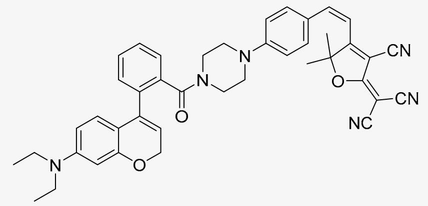	405	239	39	3min	55
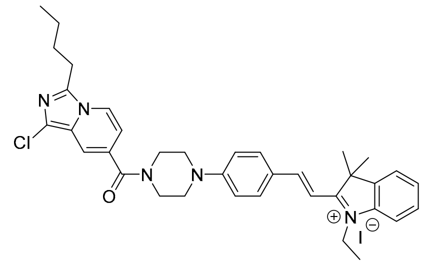	380	200	130	3min	56
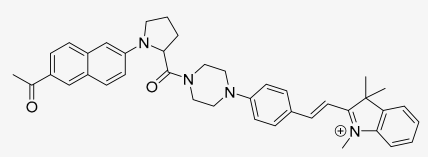	380	210	50	3min	57
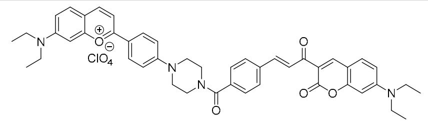	430	200	2900	4min	58
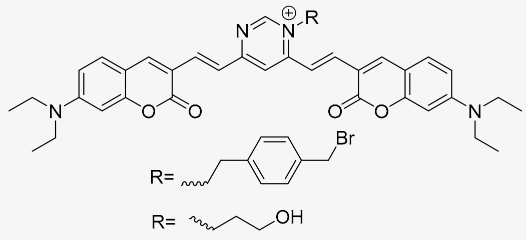	450	204	512	10min	59

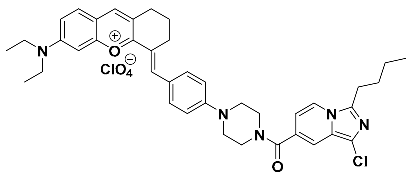	300	460	980	6s	This work

References

31. Yan, Y.; He, X.; Miao, J.; Zhao, B. A near-infrared and mitochondria-targeted fluorescence probe for ratiometric monitoring of sulfur dioxide derivatives in living cells. *J. Mater. Chem. B.* **2019**, 7, 6585–6591. https://doi.org/10.1039/C9TB01686D.
32. Shen, R.; Qian, Y. A novel ratiometric fluorescent probe for specific detection of HSO_3^- at nanomolar level through 1,4-Michael addition. *J. Photochem. Photobiol. A.* **2020**, 387, 112110. https://doi.org/10.1016/j.jphotochem.2019.112110.
33. Wu, W.; Ma, H.; Huang, M.; Miao, J.; Zhao, B. Mitochondria-targeted ratiometric fluorescent probe based on FRET for bisulfite. *Sens. Actuators, B.* **2017**, 241, 239–244. https://doi.org/10.1016/j.snb.2016.10.028.
34. Yang, D.; Ning, J.; Wu, X.; Yao, W.; Shi, H.; Miao, J.; Zhao, B.; Lin, Z. Ratiometric fluorescence sensing of endogenous sulfur dioxide derivatives: bio-imaging application in lipid droplets. *Dyes Pigm.* **2021**, 192, 109457. https://doi.org/10.1016/j.dyepig.2021.109457.
35. Li, D.; Wang, Z.; Cui, J.; Wang, X.; Miao, J.; Zhao, B. A new fluorescent probe for colorimetric and ratiometric detection of sulfur dioxide derivatives in liver cancer cells. *Sci. Rep.* **2017**, 7, 45294. https://doi.org/10.1038/srep45294.
36. Zhao, J.; Huang, L.; Yan, M.; Qu, Y.; Feng, H.; Sun, Y. A lysosome specific ratiometric fluorescent probe for detection of bisulfite ion based on hybrid coumarin-benzimidazolium compounds. *Phosphorus. Sulfur.* **2021**, 196, 321–327. https://doi.org/10.1080/10426507.2020.1835904.
37. Shen, R.; Qian, Y. A mitochondria-oriented fluorescent probe for ultrafast and ratiometric detection of HSO_3^- based on naphthalimide–hemicyanine. *New J.*

Chem. **2019**, 43, 7606–7612. https://doi.org/10.1039/C9NJ01467E.
38. Wang, Y.; Meng, Q.; Zhang, R.; Jia, H.; Wang, C.; Zhang, Z. A mitochondria-targeted ratiometric probe for the fluorescent and colorimetric detection of SO_2 derivatives in live cells. *J. Lumin.* **2017**, 192, 297–302. https://doi.org/10.1016/j.jlumin.2017.06.033.
39. Liu, K.; Chen, Y.; Sun, H.; Wang, S.; Kong, F. Construction of a novel near-infrared fluorescent probe with multiple fluorescence emission and its application for SO_2 derivative detection in cells and living zebrafish. *J. Mater. Chem. B*. **2018**, 6, 7060–7065. https://doi.org/10.1039/C8TB02030B.
40. Yang, D.; He, X.; Wu, X.; Shi, H.; Miao, J.; Zhao, B.; Lin, Z. A novel mitochondria-targeted ratiometric fluorescent probe for endogenous sulfur dioxide derivatives as a cancer-detecting tool. *J. Mater. Chem. B*. **2020**, 8, 5722–5728. https://doi.org/10.1039/D0TB00149J.
41. Yin, G.; Gan, Y.; Yu, T.; Niu, T.; Yin, P.; Chen, H.; Zhang, Y.; Li, H.; Yao, S. A dual-emission and mitochondria-targeted fluorescent probe for rapid detection of SO_2 derivatives and its imaging in living cells. *Talanta.* **2019**, 191, 428–434. https://doi.org/10.1016/j.talanta.2018.08.059.
42. Huang, Y.; Zhang, Y.; Huo, F.; Yin, C. FRET-dependent single/two-channel switch endowing a dual detection for sulfite and its organelle targeting applications. *Dyes Pigm.* **2021**, 184, 108869. https://doi.org/10.1016/j.dyepig.2020.108869.
43. Wang, M.; Liu, Q.; Sun, X.; Zheng, S.; Ma, Y.; Wang, Y.; Yan, M.; Lu, Z.; Fan, C.; Lin, W. Ratiometric and reversible detection of endogenous SO2 and HCHO in living cells and mice by a near-infrared and dual-emission fluorescent probe. *Sensors Actuators B: Chem.* **2021**, 335, 129649. https://doi.org/10.1016/j.snb.2021.129649.
44. Zhang, L.; Wang, Z.; Liu, J.; Miao, J.; Zhao, B. A rational design of ratiomet-ric fluorescent probes based on new ICT/FRET platform and imaging of endogenous sulfite in living cells. *Sens. Actuators, B.* **2017**, 253, 19–26. https://doi.org/10.1016/j.snb.2017.06.072.

45. Song, G.; Luo, J.; Xing, X.; Ma, H.; Yang, D.; Cao, X.; Ge, Y.; Zhao, B. A ratiometric fluorescence probe for rapid detection of mitochondrial SO_2 derivatives. *New J. Chem.* **2018**, 42, 3063–3068. https://doi.org/10.1039/C7NJ04021K.
46. Li, D.; Han, X.; Yan, Z.; Cui, Y.; Miao, J.; Zhao, B. A far-red ratiometric fluorescent probe for SO_2 derivatives based on the ESIPT enhanced FRET platform with improved performance. *Dyes Pigm.* **2018**, 151, 95–101. https://doi.org/10.1016/j.dyepig.2017.12.056.
47. Yan, Y.; Wu, Q.; Che, Q.; Ding, M.; Xu, M.; Miao, J.; Zhao, B.; Lin, Z. A mitochondria-targeted fluorescent probe for the detection of endogenous SO_2 derivatives in living cells. *Analyst.* **2020**, 145, 2937–2944. https://doi.org/10.1039/D0AN00086H.
48. Li, D.; Wang, Z.; Su, H.; Miao, J.; Zhao, B. Fluorescence detection of endogenous bisulfite in liver cancer cells using an effective ESIPT enhanced FRET platform. *Chem. Commun.* **2017**, 53, 577–580. https://doi.org/10.1039/C6CC06459K.
49. Lu, Y.; Dong, B.; Song, W.; Sun, Y.; Mehmood, A.; Lin, W. A mitochondria-targeting ratiometric fluorescent probe for the detection of sulfur dioxide in living cells. *New J. Chem.* **2020**, 44, 11988–11992. https://doi.org/10.1039/D0NJ02461A.
50. Li, Z.; Cui, X.; Yan, Y.; Che, Q.; Miao, J.; Zhao, B.; Lin, Z. A novel endoplasmic reticulum-targeted ratiometric fluorescent probe based on FRET for the detection of SO2 derivatives. *Dyes Pigm.* **2021**, 188, 109180. https://doi.org/10.1016/j.dyepig.2021.109180.
51. Li, D.; Wang, Z.; Cao, X.; Cui, J.; Wang, X.; Cui, H.; Miao, J.; Zhao, B. A mitochondria-targeted fluorescent probe for ratiometric detection of endogenous sulfur dioxide derivatives in cancer cells. *Chem. Commun.* **2016**, 52, 2760–2763. https://doi.org/10.1039/C5CC09092J.
52. Zhang, G.; Ji, R.; Kong, X.; Ning, F.; Liu, A.; Cui, J.; Ge, Y. A FRET based ratiometric fluorescent probe for detection of sulfite in food. *RSC Adv.* **2019**, 9,

1147–1150. https://doi.org/10.1039/C8RA08967A.

53. Yang, Y.; He, L.; Xu, K.; Lin, W. Development of a mitochondria-targeted fluorescent probe for the ratiometric visualization of sulfur dioxide in living cells and zebrafish. *Anal. Methods.* **2019**, 11, 3931–3935. https://doi.org/10.1039/C9AY01211G.
54. Xu, Z.; Chen, Z.; Liu, A.; Ji, R.; Cao, X.; Ge, Y. A ratiometric fluorescent probe for detection of exogenous mitochondrial SO2 based on a FRET mechanism. *RSC Adv.* **2019**, 9, 8943–8948. https://doi.org/10.1039/C8RA10328C.
55. Yang, X.; Zhou, Y., Zhang, X.; Yang, S.; Chen, Y.; Guo, J.; Li, X.; Qing, Z.; Yang, R. A TP-FRET-based two-photon fluorescent probe for ratiometric visualization of endogenous sulfur dioxide derivatives in mitochondria of living cells and tissues. *Chem. Commun.* **2016**, 52, 10289–10292. https://doi.org/10.1039/C6CC05254A.
56. Li, T.; Huo, F.; Chao, J.; Yin, C. Independent bi-reversible reactions and regulable FRET efficiency achieving real-time visualization of Cys metabolizing into SO_2. *Chem. Commun.* **2020**, 56, 11453–11456. https://doi.org/10.1039/D0CC04839A.
57. Chen, X.; Chen, Q.; He, D.; Yang, S.; Yang, Y.; Qian, J.; Long, L.; Wang, K. Mitochondria targeted and immobilized ratiometric NIR fluorescent probe for investigating SO2 phytotoxicity in plant mitochondria. *Sens. Actuators, B.* **2022**, 370, 132433. https://doi.org/10.1016/j.snb.2022.132433.
